# 12-(3,4,5-Tri­meth­oxy­phen­yl)-2,3,4,12-tetra­hydro-1*H*-5-oxa­tetra­phen-1-one: crystal structure and Hirshfeld surface analysis

**DOI:** 10.1107/S2056989016007775

**Published:** 2016-05-13

**Authors:** Mukesh M. Jotani, P. Iniyavan, V. Vijayakumar, S. Sarveswari, Yee Seng Tan, Edward R. T. Tiekink

**Affiliations:** a Department of Physics, Bhavan’s Sheth R. A. College of Science, Ahmedabad, Gujarat 380 001, India; b Centre for Organic and Medicinal Chemistry, VIT University, Vellore, Tamil Nadu 632 014, India; c Research Centre for Crystalline Materials, Faculty of Science and Technology, Sunway University, 47500 Bandar Sunway, Selangor Darul Ehsan, Malaysia

**Keywords:** crystal structure, xanthene, conformation, Hirshfeld surface

## Abstract

In the title compound, C_26_H_24_O_5_, the pyran ring has a flattened-boat con­formation, with the 1,4-related ether O and methine C atoms lying 0.1205 (18) and 0.271 (2) Å, respectively, above the least-squares plane involving the doubly bonded C atoms (r.m.s deviation = 0.0208 Å). An envelope conformation is found for the cyclo­hexene ring, with the flap atom being the middle methyl­ene C atom, lying 0.616 (2) Å out of the plane defined by the remaining atoms (r.m.s. deviation = 0.0173 Å). The fused four-ring system is approximately planar, with the dihedral angle between the least-squares planes through the cyclo­hexene and naphthyl rings being 10.78 (7)°. The tris­ubstituted benzene ring occupies a position almost perpendicular to the pyran ring [dihedral angle = 83.97 (4)°]. The most prominent feature of the packing is the formation of zigzag supra­molecular chains mediated by aryl-C—H⋯O(meth­oxy) inter­actions; chains are connected into a three-dimensional architecture by methyl­ene- and methyl-C—H⋯π inter­actions. The prevalence of C—H⋯O and C—H⋯π inter­actions is confirmed by an analysis of the Hirshfeld surface. A comparison with related structures suggests that the mol­ecular conformation of the title compound is relatively robust with respect to varying substitution patterns at the methine C atom of the pyran ring.

## Abstract 2

The pyran and cyclo­hexene rings of the title compound adopt flattened-boat and envelope conformations, respectively. In the crystal, zigzag supra­molecular chains are formed *via* aryl-*C*—*H*⋯*O*(meth­oxy) inter­actions.

## Chemical context   

Xanthenes and benzoxanthenes are important bioactive compounds that possess a wide range of biological and thera­peutic properties, such as analgesic (Hafez *et al.*, 2008 ▸), anti­viral and anti­bacterial and anti-inflammatory activities (Poupelin *et al.*, 1978 ▸; Hideo & Teruomi, 1981 ▸; Asano *et al.*, 1996 ▸; Matsumoto *et al.*, 2005 ▸; Pinto *et al.*, 2005 ▸; Woo *et al.*, 2007 ▸; Pouli & Marakos, 2009 ▸). Some of these compounds have been used in photodynamic therapy (Ion, 1997 ▸). Further, due to their having desirable spectroscopic properties, some derivatives have been used as dyes in laser technologies (Menchen *et al.*, 2003 ▸) and as pH-sensitive fluorescent materials for the visualization of biomolecules (Ahmad *et al.*, 2002 ▸).

Various methods for the synthesis of tetra­hydro­benzo[*a*]xanthen-11-ones have been reported (Knight & Stephens, 1989 ▸). These usually involve a three-component condensation of dimedone with an aromatic aldehyde and 2-naphthol. However, each of these procedures has some drawbacks, such as harsh reaction conditions, tedious work-up and low yields. Hence, the microwave-assisted ionic liquid-mediated synthesis of xanthenes from cyclo­hexane-1,3-dione, 3,4,5-tri­meth­oxy­benzaldehyde and 2-naphthol was attempted. The use of an ionic liquid, *i.e*. [1-butyl-3-methyl­imid­azol­ium]­PF_6_, and microwave irradiation afforded the title compound in high yield within 12 min (Iniyavan *et al.*, 2015 ▸). The title compound is a potent anti-oxidant (Iniyavan *et al.*, 2015 ▸) and herein its crystal and mol­ecular structures are described, along with an analysis of its Hirshfeld surface in order to gain greater insight into the crystal packing, especially the role of weaker inter­actions.
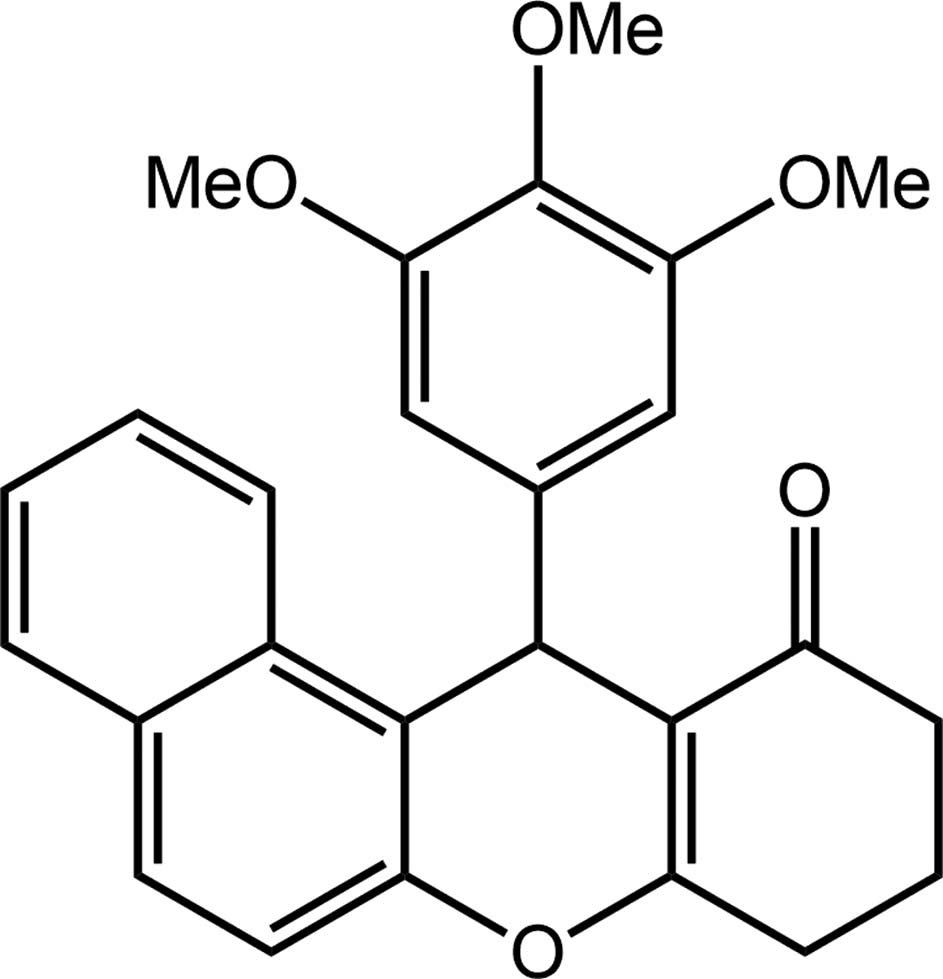


## Structural commentary   

**Figure 1:**
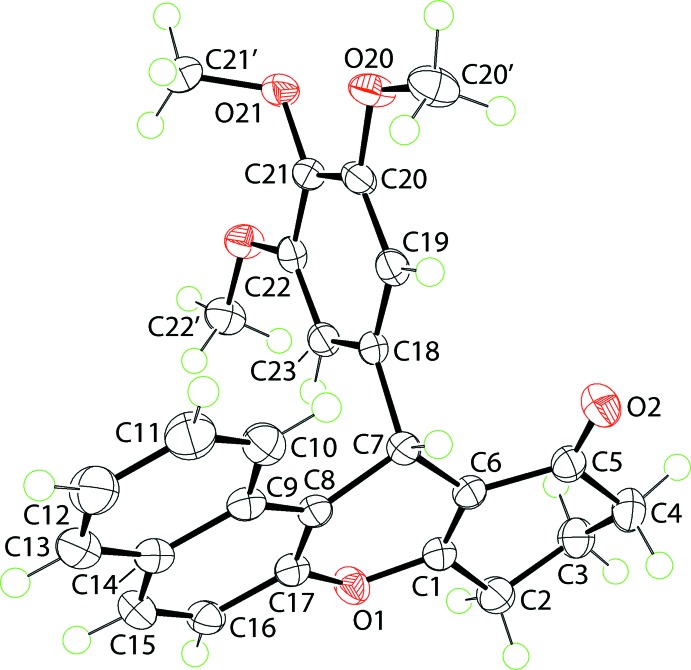
The mol­ecular structure of (I), showing the atom-labelling scheme and displacement ellipsoids at the 70% probability level.

The central pyran ring in the title compound, (I), is flanked by both a cyclo­hexene ring and a naphthyl-fused ring system (Fig. 1 ▸). A tris­ubstituted benzene ring is connected to the aforementioned four-ring residue at the methine C7 atom. The pyran ring has a flattened boat conformation, with the 1,4-related O1 and C7 atoms lying 0.1205 (18) and 0.271 (2) Å to the same side of the plane (r.m.s. deviation of the fitted atoms = 0.0208 Å) defined by the C1=C6 [1.3431 (19) Å] and C8=C17 [1.3681 (19) Å] double bonds. To a first approximation, the cyclo­hexene ring has an envelope conformation, with the C3 (flap) atom lying 0.616 (2) Å above the plane defined by the remaining atoms (r.m.s. deviation = 0.0173 Å). The atoms comprising the four-ring system are approximately coplanar, as seen in the dihedral angle between the best plane through the cyclo­hexene ring and naphthyl residue of 10.78 (7)°. The benzene ring occupies a position almost perpendicular to the previous residue, forming a dihedral angle of 83.97 (4)° with the best plane through the pyran ring. In the benzene ring, two meth­oxy groups are coplanar with the ring to which they are connected [the C20′—O20—C20—C19 and C22′—O22—C22—C23 torsion angles are 4.98 (19) and 0.51 (19)°, respectively], while the central substituent is approximately perpendicular to the ring lying over the naphthyl residue, *i.e*. C21′—O21—C21—C22 is 76.08 (16)°. Presumably, this conformation is adopted to reduce steric hindrance.

## Supra­molecular features   

**Figure 2:**
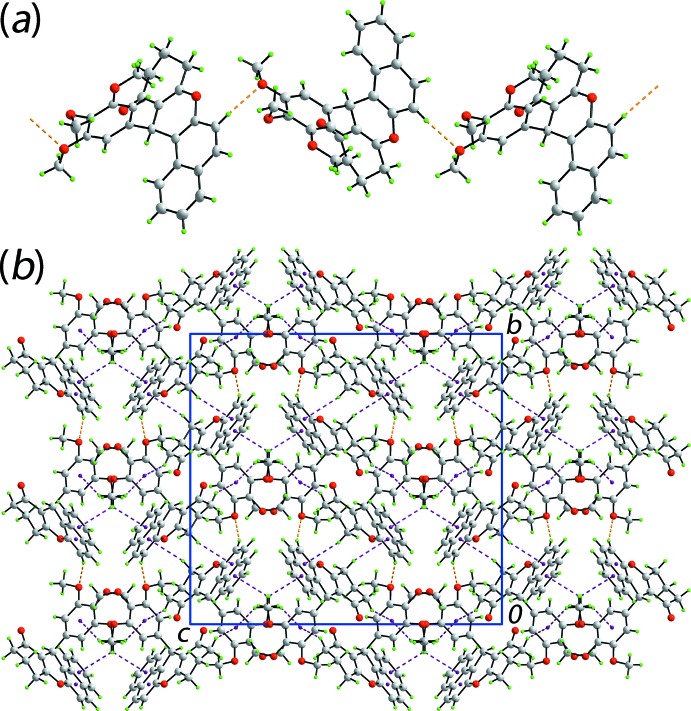
The mol­ecular packing in (I): (*a*) a view of the supra­molecular chain along the *a* axis sustained by C—H⋯O inter­actions shown as orange dashed lines and (*b*) the unit-cell contents shown in projection down the *a* axis with the C—H⋯π(ar­yl) inter­actions shown as purple dashed lines.

In the mol­ecular packing of (I), supra­molecular chains along the *a* axis are formed through the agency of relatively strong aryl-C16—H16⋯O(meth­oxy) inter­actions (Table 1 ▸). Being generated by glide symmetry, the topology of the chain is zigzag (Fig. 2 ▸ *a*). The chains are connected into a three-dimensional architecture by a network of C—H⋯π(ar­yl) inter­actions (Table 1 ▸). The donor atoms are derived from methyl­ene and methyl groups, with the acceptor rings being each of the aromatic rings and with the outer benzene ring participating in two such contacts (Fig. 2 ▸ *b*).

## Hirshfeld surface analysis   

With the aid of the program *Crystal Explorer* (Wolff *et al.*, 2012 ▸), Hirshfeld surfaces mapped over *d*_norm_, *d*_e_, curvedness and electrostatic potential were generated. The electrostatic potential was calculated with *TONTO* (Spackman *et al.*, 2008 ▸; Jayatilaka *et al.*, 2005 ▸), integrated in *Crystal Explorer*, using the crystal structure as the starting geometry. The electrostatic potentials were mapped on the Hirshfeld surface using the STO-3G basis/Hartree–Fock level of theory over the range ±0.08 au. The contact distances *d*_i_ and *d*_e_ from the Hirshfeld surface to the nearest atom inside and outside, respectively, enables the analysis of the inter­molecular inter­actions through the mapping of *d*_norm_. The combination of *d*_e_ and *d*_i_ in the form of a two-dimensional fingerprint plot (McKinnon *et al.*, 2004 ▸) provides a convenient summary of the inter­molecular contacts in the crystal.

**Figure 3:**
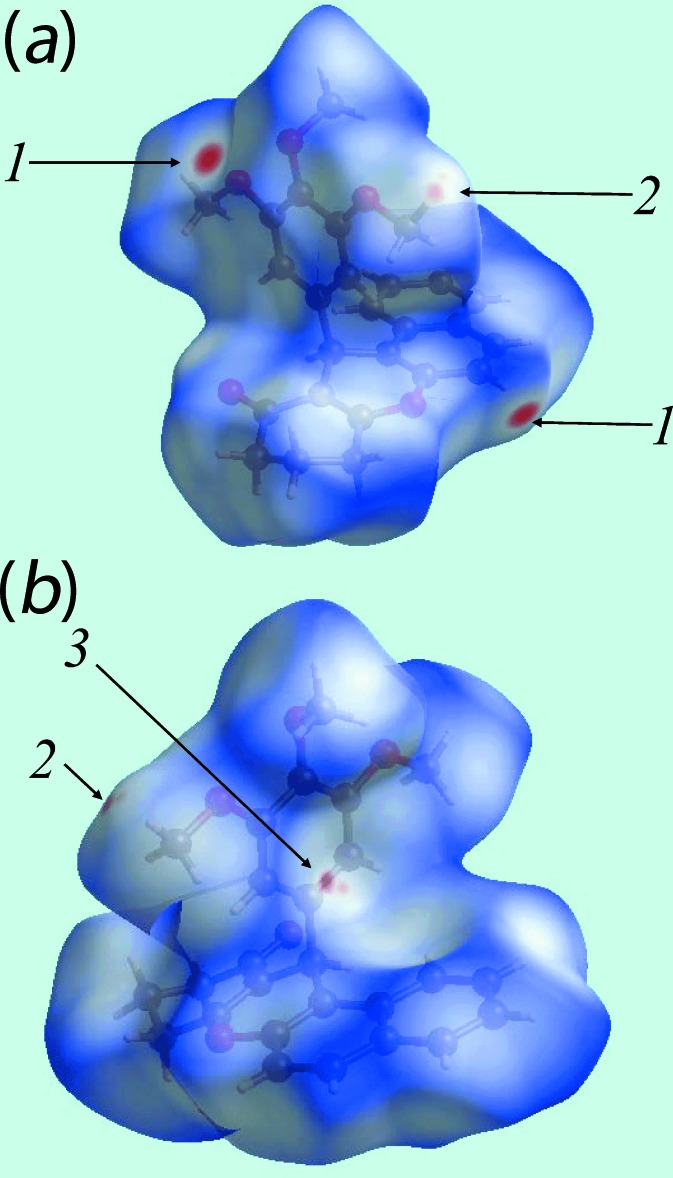
Two views of Hirshfeld surfaces mapped over *d*_norm_ for (I). Labels ‘**1**’, ‘**2**’ and ‘**3**’ indicate specific inter­molecular inter­actions (see text).

**Figure 4:**
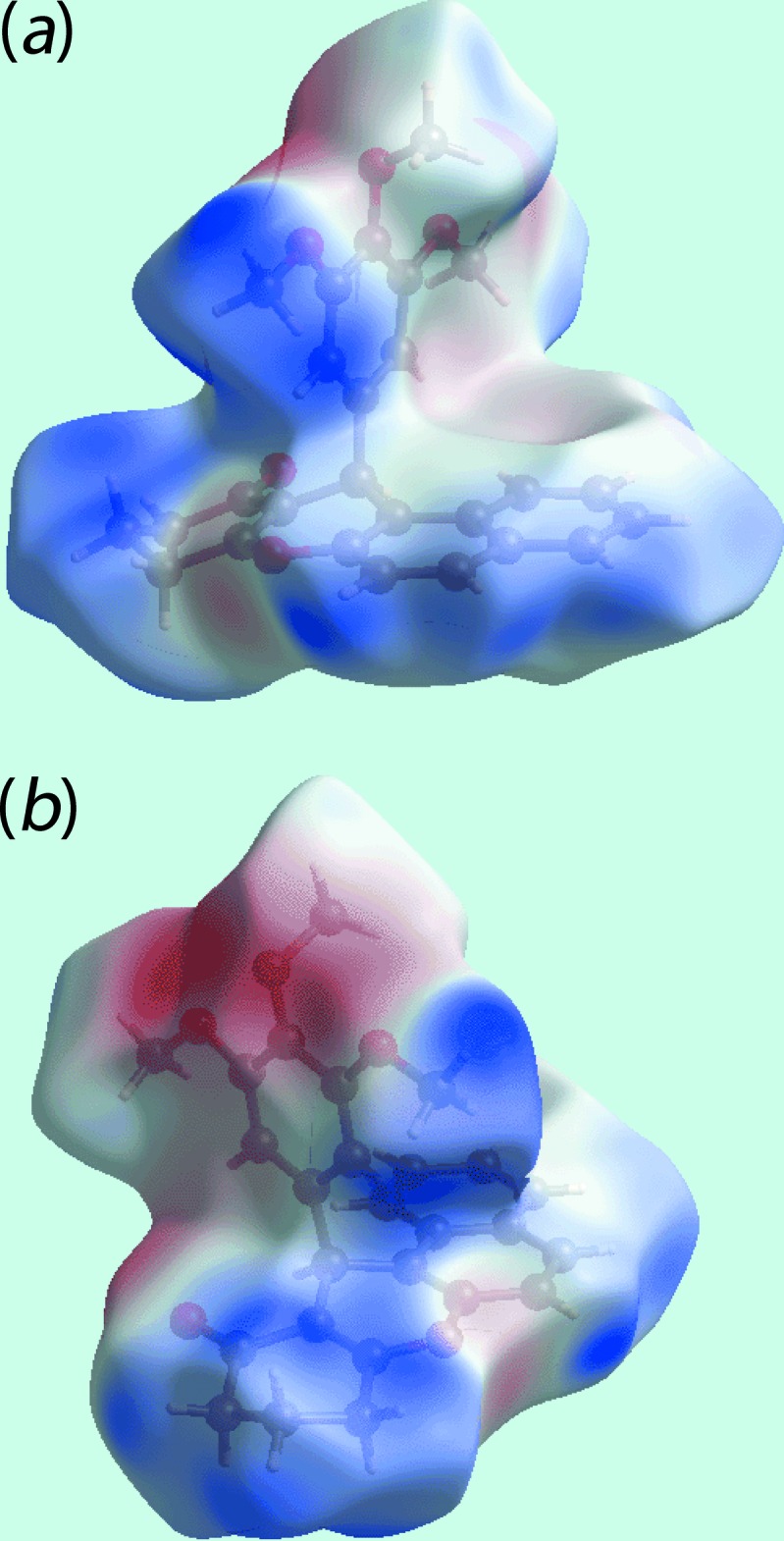
Two views of Hirshfeld surfaces mapped over electrostatic potential for (I). The red and blue regions represent negative and positive electrostatic potentials, respectively.

The bright-red spots at the aryl H16 and meth­oxy O20 atoms, visible on the Hirshfeld surface mapped over *d*_norm_ and labelled as ‘**1**’ in Fig. 3 ▸, represent the donor and acceptor atoms for the inter­molecular C—H⋯O inter­action, respectively. On the surface mapped over electrostatic potential (Fig. 4 ▸), these inter­actions appear as the respective blue and red regions. The views of surfaces mapped over *d*_norm_, *d*_e_, electrostatic potential and shape-index (Figs. 3 ▸–6 ▸ ▸ ▸) highlight the significant role of C—H⋯π inter­actions in the packing. In particular, the involvement of the meth­oxy C22′—H group in two C—H⋯π inter­actions with the symmetry-related aryl rings (Table 1 ▸) are evident from the two faint-red spots near these atoms on the *d*_norm_ mapped surface, indicated with ‘**2**’ in Fig. 3 ▸.

**Figure 5:**
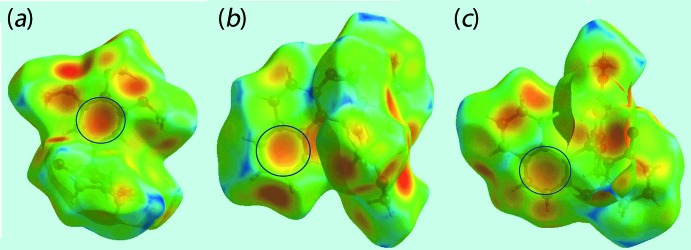
Views of Hirshfeld surface mapped over *d*_e_ for (I). The pale-orange spots within blue circles indicate the involvement of aryl ring atoms in C—H⋯π inter­actions.

**Figure 6:**
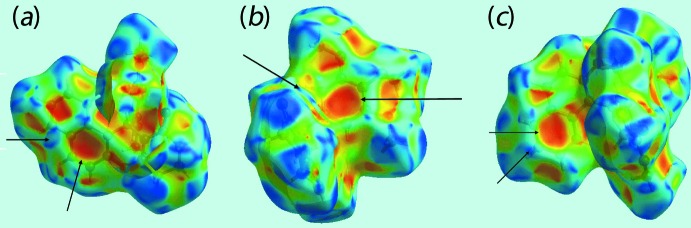
Views of Hirshfeld surface mapped with the shape-index property for (I). The bright-red spots identified with arrows indicate the C—H⋯π inter­actions, while the blue spots indicate complementary π⋯H—C inter­actions.

The corresponding regions on the Hirshfeld surface mapped over electrostatic potential (Fig. 4 ▸) appear as blue and light-red, respectively. The remaining C—H⋯π inter­actions, involving the methyl­ene H2*B* and H4*B* atoms as donors, and the C8/C9/C14–C17 and C18–C23 rings as π-acceptors, are also evident from Fig. 4 ▸, through the appearance of respective blue and light-red regions near these atoms. The network of these C—H⋯π inter­actions are also recognized through the pale-orange spots present on the Hirshfeld surfaces mapped over *d*_e_, highlighted within blue circles in Fig. 5 ▸, and as bright-red spots over the front side of shape-indexed surfaces identified with arrows in Fig. 6 ▸. The reciprocal of these C—H⋯π inter­actions, *i.e*. π⋯H—C, are also seen as blue spots on the shape-indexed surface in Fig. 6 ▸. The faint-red spots near the phenyl C23 atom on the surface mapped over *d*_norm_, labelled as ‘**3**’ in Fig. 3 ▸, indicate the presence of short interatomic C⋯H/H⋯C contacts in the crystal, Table 2 ▸.

**Figure 7:**
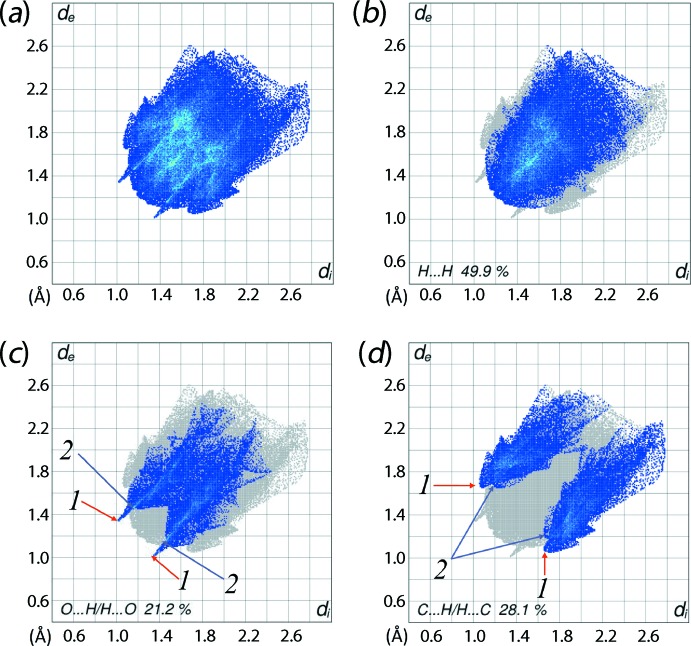
Two-dimensional fingerprint plots calculated for (I): (*a*) overall plot, and those delineated into (*b*) H⋯H, (*c*) O⋯H/H⋯O and (*d*) C⋯H/H⋯C contacts.

The overall two-dimensional fingerprint plot (Fig. 7 ▸ *a*) and those delineated (McKinnon *et al.*, 2007 ▸) into H⋯H, O⋯H/H⋯O and C⋯H/H⋯C contacts are illustrated in Figs. 7 ▸(*b*–*d*), respectively; their relative contributions are summarized in Table 3 ▸. The inter­atomic H⋯H contacts at distances greater than their van der Waals separation appear as scattered points in the greater part of the fingerprint plot (Fig. 7 ▸ *b*), and makes the most significant contribution to the overall Hirshfeld surface, *i.e*. 49.3%. In the fingerprint plot delineated into O⋯H/H⋯O contacts, a pair of short spikes at *d*_e_ + *d*_i_ ∼ 2.4 Å, and the cluster of blue points aligned in pairs with (*d*_e_ + *d*_i_)_min_ ∼ 2.7 Å, identified with labels ‘**1**’ and ‘**2**’, respectively, in Fig. 7 ▸(*c*), corresponds to a 21.2% contribution to the Hirshfeld surface. These features reflect the presence of aryl-C16—H16⋯O(meth­oxy) inter­actions, as well as the short inter­atomic O⋯H/H⋯O contacts between carboxyl O2 and methyl­ene H3*B* atoms (Table 2 ▸).

The fingerprint plot delineated into C⋯H/H⋯C contacts, with a 28.1% contribution to the Hirshfeld surface, shows the points in the plot arranged in the form of two pairs of arrow-like shapes with their tips at *d*_e_ + *d*_i_ = 2.70 and 2.85 Å, labelled as ‘**1**’ and ‘**2**’ in Fig. 7 ▸(*d*), respectively. These features reflect the presence of C—H⋯π inter­actions and short inter­atomic C⋯H/H⋯C contacts (Table 3 ▸) in the crystal. The absence of π–π stacking inter­actions is consistent with their being no contribution from C⋯C contacts to the Hirshfeld surface (Table 3 ▸).

The final analysis of the mol­ecular packing involves a relatively new descriptor, *i.e*. the enrichment ratio (ER) (Jelsch *et al.*, 2014 ▸); data are collated in Table 4 ▸. The involvement of surface H atoms in C—H⋯π inter­actions and the presence of a number of inter­atomic C⋯H contacts (Table 3 ▸) yields an ER value for H⋯H contacts less than unity, *i.e*. 0.90. The presence of these inter­actions explains the enhanced ER value of 1.31 for C⋯H/H⋯C contacts, consistent with their high propensity to form in the mol­ecular packing of (I). The O atoms comprise only 11.1% of the surface but provide a 21.2% contribution from O⋯H/H⋯O contacts to the Hirshfeld surface. Reflecting this, the ER value is 1.28, which is in the expected 1.2–1.6 range. Other contacts, namely C⋯C, O⋯O and C⋯O/O⋯C, show no propensity to form as reflected in their low ER values (Table 4 ▸).

## Database survey   

**Figure 8:**
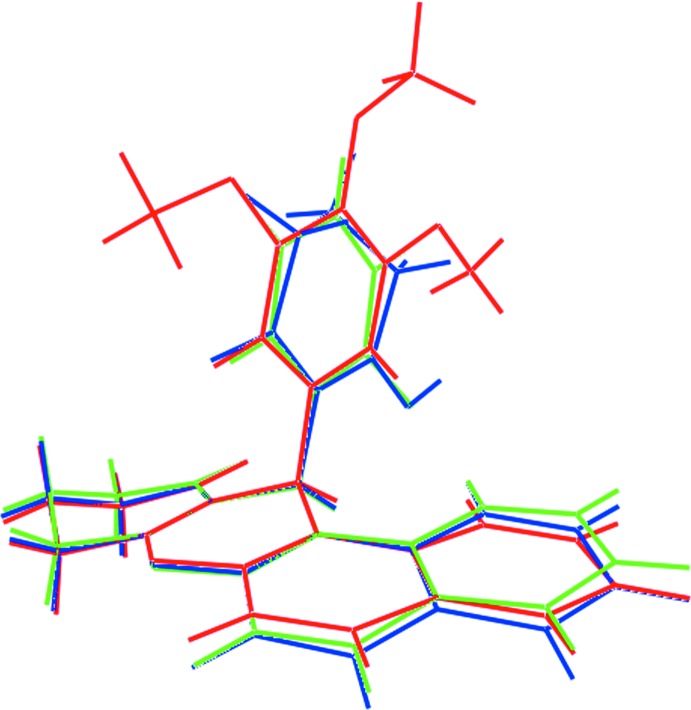
Overlap diagram of the title compound, (I) (red image), with literature precedents (II) (green) and (III) (blue). The mol­ecules have been overlapped so that the C1, C6 and C8 atoms are coincident.

There are two structures in the crystallographic literature (Groom *et al.*, 2016 ▸) featuring the methine-substituted 2,3,4,12-tetra­hydro-5-oxa­tetra­phen-1-one residue, as in (I). In the most closely related structure, (II) (Sethukumar *et al.*, 2012 ▸), with a 2-chloro­benzene ring at the methine C7 atom, an essentially similar conformation is found, as emphasized in the overlay diagram shown in Fig. 8 ▸. Here, the dihedral angle between the best plane through the cyclo­hexene ring and naphthyl residue is 7.50 (6)°, *i.e.* marginally less folded than in (I) where the angle was 10.78 (7)°. The angle between the least-squares planes through the pyran and benzene rings is 89.71 (6)°. Despite having a bulky 2-hy­droxy-6-oxo­cyclo­hex-1-enyl residue at the methine C7 atom, rather than an aryl ring, the conformation in (III) (Akkurt *et al.*, 2013 ▸) bears a close resemblance to those of (I) and (II). Thus, in (III), the cyclo­hexene/naphthyl dihedral angle is 16.26 (5)°, indicating a non-folded four-ring residue, and the pyran/cyclohexenyl dihedral angle is 85.57 (6)°. Clearly, the non-folded conformation of the 2,3,4,12-tetra­hydro-5-oxa­tetra­phen-1-one core and its orthogonal relationship to the methine C7-bound substituent in (I)–(III) is to a first robust.

## Synthesis and crystallization   

The title compound was prepared and characterized spectroscopically as per the literature (Iniyavan *et al.*, 2015 ▸). Crystals for the X-ray study were obtained after 2 d of slow evaporation of a chloro­form solution of (I) held at room temperature.

## Refinement details   

Crystal data, data collection and structure refinement details are summarized in Table 5 ▸. Carbon-bound H atoms were placed in calculated positions (C—H = 0.95–1.00 Å) and were included in the refinement in the riding model approximation, with *U*_iso_(H) set at 1.2–1.5*U*_eq_(C).

## Supplementary Material

Crystal structure: contains datablock(s) I, global. DOI: 10.1107/S2056989016007775/hb7584sup1.cif

Structure factors: contains datablock(s) I. DOI: 10.1107/S2056989016007775/hb7584Isup2.hkl

Supporting information file. DOI: 10.1107/S2056989016007775/hb7584Isup3.cml

CCDC reference: 1479203

Additional supporting information:  crystallographic information; 3D view; checkCIF report

## Figures and Tables

**Table 1:** Hydrogen-bond geometry (Å, °) *Cg*1, *Cg*2 and *Cg*3 are the centroids of the C8/C9/C14–C17, C18–C23 and C9–C14 rings, respectively.

*D*—H⋯*A*	*D*—H	H⋯*A*	*D*⋯*A*	*D*—H⋯*A*
C16—H16⋯O20^i^	0.95	2.36	3.2604 (18)	159
C2—H2*B*⋯*Cg*1^ii^	0.99	2.92	3.8088 (16)	150
C4—H4*B*⋯*Cg*2^iii^	0.99	2.75	3.5605 (16)	140
C22′—H22*B*⋯*Cg*2^iv^	0.98	2.56	3.3918 (16)	143
C22′—H22*C*⋯*Cg*3^iv^	0.98	2.78	3.4332 (16)	125

Symmetry codes: (i) 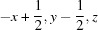; (ii) 
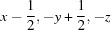
; (iii) 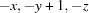; (iv) 
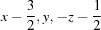
.

**Table 2:** Additional short inter­atomic contacts (Å) for the title compound

Inter­action	Distance	Symmetry operation
C23⋯H4*B*	2.86	−*x*, 1 − *y*, −*z*
C23⋯H22*B*	2.72	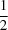 + *x*, *y*, 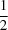 − *z*
C11⋯H2*A*	2.86	1 + *x*, *y*, *z*
C11⋯H22*A*	2.83	1 + *x*, *y*, *z*
O2⋯H3*B*	2.61	−*x*, 1 − *y*, −*z*
C12⋯H22*A*	2.82	1 + *x*, *y*, *z*
C18⋯H22*B*	2.77	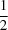 + *x*, *y*, 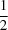 − *z*
C22⋯H22*B*	2.86	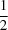 + *x*, *y*, 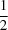 − *z*
C21′⋯H4*A*	2.82	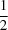 − *x*, 1 − *y*, 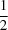 + *z*

**Table 3:** Percentage contribution of the different inter­molecular inter­actions to the Hirshfeld surface of (I)

Contact	Contribution
H⋯H	49.9
O⋯H/H⋯O	21.2
C⋯H/H⋯C	28.1
C⋯O/O⋯C	0.6
O⋯O	0.2
C⋯C	0.0

**Table 4:** Enrichment ratios (ER) for the title compound

Inter­action	ER
H⋯H	0.90
O⋯H/H⋯O	1.28
C⋯H/H⋯C	1.31
C⋯C	0.0
C⋯O/O⋯C	0.19
O⋯O	0.16

**Table 5:** Experimental details

Crystal data	
Chemical formula	C_26_H_24_O_5_
*M*_r_	416.45
Crystal system, space group	Orthorhombic, *P* *b* *c* *a*
Temperature (K)	100
*a*, *b*, *c* (Å)	9.2164 (5), 20.3760 (9), 21.8731 (9)
*V* (Å^3^)	4107.6 (3)
*Z*	8
Radiation type	Mo *K*α
μ (mm^−1^)	0.09
Crystal size (mm)	0.25 × 0.20 × 0.20

Data collection	
Diffractometer	Agilent Technologies SuperNova Dual diffractometer with an Atlas detector
Absorption correction	Multi-scan (*CrysAlis PRO*; Agilent, 2014 ▸)
*T*_min_, *T*_max_	0.855, 1.000
No. of measured, independent and observed [*I* > 2σ(*I*)] reflections	23597, 4664, 3991
*R*_int_	0.034
(sin θ/λ)_max_ (Å^−1^)	0.650

Refinement	
*R*[*F* ^2^ > 2σ(*F* ^2^)], *wR*(*F* ^2^), *S*	0.040, 0.100, 1.04
No. of reflections	4664
No. of parameters	283
H-atom treatment	H-atom parameters constrained
Δρ_max_, Δρ_min_ (e Å^−3^)	0.34, −0.22

Computer programs: *CrysAlis PRO* (Agilent, 2014 ▸), *SHELXS97* (Sheldrick, 2008 ▸), *SHELXL2014* (Sheldrick, 2015 ▸), *ORTEP-3 for Windows* (Farrugia, 2012 ▸), *QMol* (Gans & Shalloway, 2001 ▸), *DIAMOND* (Brandenburg, 2006 ▸) and *publCIF* (Westrip, 2010 ▸).

## supplementary crystallographic information

### Crystal data

**Table:**

C_26_H_24_O_5_	*D*_x_ = 1.347 Mg m^−^^3^
*M**_r_* = 416.45	Mo *K*α radiation, λ = 0.71073 Å
Orthorhombic, *P**b**c**a*	Cell parameters from 7968 reflections
*a* = 9.2164 (5) Å	θ = 3.5–29.3°
*b* = 20.3760 (9) Å	µ = 0.09 mm^−^^1^
*c* = 21.8731 (9) Å	*T* = 100 K
*V* = 4107.6 (3) Å^3^	Prism, colourless
*Z* = 8	0.25 × 0.20 × 0.20 mm
*F*(000) = 1760	

### Data collection

**Table:**

Agilent Technologies SuperNova Dual diffractometer with an Atlas detector	4664 independent reflections
Radiation source: SuperNova (Mo) X-ray Source	3991 reflections with *I* > 2σ(*I*)
Mirror monochromator	*R*_int_ = 0.034
Detector resolution: 10.4041 pixels mm^-1^	θ_max_ = 27.5°, θ_min_ = 2.9°
ω scan	*h* = −9→11
Absorption correction: multi-scan (*CrysAlis PRO*; Agilent, 2014)	*k* = −26→24
*T*_min_ = 0.855, *T*_max_ = 1.000	*l* = −28→25
23597 measured reflections	

### Refinement

**Table:**

Refinement on *F*^2^	0 restraints
Least-squares matrix: full	Hydrogen site location: inferred from neighbouring sites
*R*[*F*^2^ > 2σ(*F*^2^)] = 0.040	H-atom parameters constrained
*wR*(*F*^2^) = 0.100	*w* = 1/[σ^2^(*F*_o_^2^) + (0.0327*P*)^2^ + 3.0207*P*] where *P* = (*F*_o_^2^ + 2*F*_c_^2^)/3
*S* = 1.04	(Δ/σ)_max_ < 0.001
4664 reflections	Δρ_max_ = 0.34 e Å^−^^3^
283 parameters	Δρ_min_ = −0.22 e Å^−^^3^

### Special details

**Table:**

Geometry. All esds (except the esd in the dihedral angle between two l.s. planes) are estimated using the full covariance matrix. The cell esds are taken into account individually in the estimation of esds in distances, angles and torsion angles; correlations between esds in cell parameters are only used when they are defined by crystal symmetry. An approximate (isotropic) treatment of cell esds is used for estimating esds involving l.s. planes.

### Fractional atomic coordinates and isotropic or equivalent isotropic displacement parameters (Å^2^)

**Table:**

	*x*	*y*	*z*	*U*_iso_*/*U*_eq_	
O1	0.13499 (11)	0.29394 (5)	0.08369 (4)	0.0202 (2)	
O2	0.21599 (12)	0.47795 (5)	−0.04262 (5)	0.0249 (2)	
O20	0.43308 (11)	0.62718 (5)	0.14235 (4)	0.0215 (2)	
O21	0.24076 (11)	0.61119 (5)	0.23202 (4)	0.0204 (2)	
O22	0.09183 (11)	0.49986 (5)	0.24461 (4)	0.0202 (2)	
C1	0.10078 (15)	0.33649 (7)	0.03771 (6)	0.0181 (3)	
C2	−0.03608 (16)	0.31688 (7)	0.00588 (7)	0.0223 (3)	
H2A	−0.1058	0.2993	0.0362	0.027*	
H2B	−0.0146	0.2817	−0.0240	0.027*	
C3	−0.10412 (16)	0.37501 (7)	−0.02719 (7)	0.0236 (3)	
H3A	−0.1826	0.3593	−0.0544	0.028*	
H3B	−0.1473	0.4054	0.0031	0.028*	
C4	0.00918 (17)	0.41132 (8)	−0.06483 (6)	0.0249 (3)	
H4A	0.0396	0.3831	−0.0994	0.030*	
H4B	−0.0354	0.4514	−0.0822	0.030*	
C5	0.14184 (16)	0.43053 (7)	−0.02839 (6)	0.0188 (3)	
C6	0.18328 (15)	0.38862 (6)	0.02343 (6)	0.0172 (3)	
C7	0.31954 (15)	0.40554 (6)	0.05870 (6)	0.0159 (3)	
H7	0.3968	0.4186	0.0290	0.019*	
C8	0.37084 (15)	0.34558 (6)	0.09372 (6)	0.0163 (3)	
C9	0.51546 (15)	0.34033 (6)	0.11767 (6)	0.0179 (3)	
C10	0.62592 (16)	0.38608 (7)	0.10377 (6)	0.0215 (3)	
H10	0.6052	0.4217	0.0772	0.026*	
C11	0.76293 (17)	0.37994 (8)	0.12805 (7)	0.0261 (3)	
H11	0.8356	0.4111	0.1179	0.031*	
C12	0.79633 (17)	0.32772 (8)	0.16789 (7)	0.0280 (3)	
H12	0.8914	0.3236	0.1842	0.034*	
C13	0.69162 (18)	0.28293 (7)	0.18297 (7)	0.0267 (3)	
H13	0.7142	0.2483	0.2104	0.032*	
C14	0.55010 (16)	0.28747 (7)	0.15820 (6)	0.0207 (3)	
C15	0.44087 (16)	0.24150 (7)	0.17401 (7)	0.0225 (3)	
H15	0.4618	0.2079	0.2028	0.027*	
C16	0.30631 (16)	0.24481 (7)	0.14842 (6)	0.0196 (3)	
H16	0.2349	0.2127	0.1578	0.024*	
C17	0.27480 (15)	0.29668 (7)	0.10782 (6)	0.0175 (3)	
C18	0.29511 (14)	0.46231 (6)	0.10361 (6)	0.0153 (3)	
C19	0.37542 (15)	0.51971 (6)	0.09866 (6)	0.0168 (3)	
H19	0.4417	0.5253	0.0658	0.020*	
C20	0.35832 (15)	0.56922 (6)	0.14226 (6)	0.0168 (3)	
C20'	0.52664 (17)	0.63990 (7)	0.09194 (7)	0.0244 (3)	
H20A	0.6011	0.6056	0.0897	0.037*	
H20B	0.5732	0.6827	0.0974	0.037*	
H20C	0.4700	0.6401	0.0540	0.037*	
C21	0.26077 (15)	0.56129 (6)	0.19031 (6)	0.0166 (3)	
C21'	0.30768 (18)	0.59803 (7)	0.29015 (6)	0.0255 (3)	
H21A	0.2686	0.5571	0.3070	0.038*	
H21B	0.2872	0.6342	0.3183	0.038*	
H21C	0.4128	0.5938	0.2847	0.038*	
C22	0.18044 (15)	0.50332 (6)	0.19494 (6)	0.0162 (3)	
C22'	0.01074 (17)	0.44121 (7)	0.25262 (7)	0.0225 (3)	
H22A	−0.0589	0.4366	0.2190	0.034*	
H22B	−0.0416	0.4431	0.2916	0.034*	
H22C	0.0768	0.4035	0.2528	0.034*	
C23	0.19668 (15)	0.45412 (6)	0.15136 (6)	0.0165 (3)	
H23	0.1408	0.4151	0.1542	0.020*	

### Atomic displacement parameters (Å^2^)

**Table:**

	*U*^11^	*U*^22^	*U*^33^	*U*^12^	*U*^13^	*U*^23^
O1	0.0199 (5)	0.0172 (5)	0.0234 (5)	−0.0026 (4)	−0.0031 (4)	0.0040 (4)
O2	0.0296 (6)	0.0215 (5)	0.0237 (5)	−0.0001 (4)	0.0005 (4)	0.0052 (4)
O20	0.0258 (6)	0.0131 (4)	0.0255 (5)	−0.0026 (4)	0.0055 (4)	−0.0010 (4)
O21	0.0282 (6)	0.0143 (5)	0.0187 (5)	0.0031 (4)	0.0013 (4)	−0.0022 (4)
O22	0.0232 (5)	0.0180 (5)	0.0196 (5)	−0.0009 (4)	0.0058 (4)	−0.0012 (4)
C1	0.0210 (7)	0.0167 (6)	0.0167 (6)	0.0021 (5)	−0.0013 (5)	−0.0011 (5)
C2	0.0225 (7)	0.0202 (7)	0.0242 (7)	−0.0009 (6)	−0.0043 (6)	−0.0004 (5)
C3	0.0222 (7)	0.0250 (7)	0.0237 (7)	0.0020 (6)	−0.0055 (6)	0.0003 (6)
C4	0.0287 (8)	0.0276 (7)	0.0183 (7)	0.0026 (6)	−0.0047 (6)	0.0022 (6)
C5	0.0223 (7)	0.0190 (6)	0.0151 (6)	0.0042 (5)	0.0019 (5)	−0.0009 (5)
C6	0.0205 (7)	0.0168 (6)	0.0142 (6)	0.0023 (5)	0.0006 (5)	−0.0013 (5)
C7	0.0183 (7)	0.0141 (6)	0.0153 (6)	−0.0003 (5)	0.0004 (5)	0.0005 (5)
C8	0.0202 (7)	0.0147 (6)	0.0140 (6)	0.0015 (5)	0.0010 (5)	−0.0016 (5)
C9	0.0206 (7)	0.0157 (6)	0.0173 (6)	0.0015 (5)	0.0001 (5)	−0.0032 (5)
C10	0.0224 (7)	0.0203 (7)	0.0219 (7)	0.0005 (6)	0.0015 (6)	0.0008 (5)
C11	0.0244 (8)	0.0258 (7)	0.0282 (7)	−0.0028 (6)	−0.0001 (6)	−0.0013 (6)
C12	0.0233 (8)	0.0271 (8)	0.0335 (8)	0.0015 (6)	−0.0082 (7)	−0.0032 (6)
C13	0.0316 (8)	0.0188 (7)	0.0295 (8)	0.0028 (6)	−0.0068 (7)	−0.0012 (6)
C14	0.0249 (7)	0.0158 (6)	0.0214 (6)	0.0016 (5)	−0.0035 (6)	−0.0026 (5)
C15	0.0279 (8)	0.0167 (6)	0.0229 (7)	0.0027 (6)	−0.0017 (6)	0.0029 (5)
C16	0.0242 (7)	0.0147 (6)	0.0199 (6)	−0.0021 (5)	0.0008 (6)	0.0001 (5)
C17	0.0193 (7)	0.0161 (6)	0.0170 (6)	0.0013 (5)	−0.0012 (5)	−0.0019 (5)
C18	0.0174 (6)	0.0143 (6)	0.0142 (6)	0.0023 (5)	−0.0035 (5)	0.0007 (5)
C19	0.0183 (7)	0.0161 (6)	0.0159 (6)	0.0012 (5)	0.0007 (5)	0.0024 (5)
C20	0.0182 (7)	0.0120 (6)	0.0202 (6)	0.0001 (5)	−0.0018 (5)	0.0027 (5)
C20'	0.0243 (8)	0.0181 (7)	0.0308 (8)	−0.0026 (6)	0.0075 (6)	0.0012 (6)
C21	0.0200 (7)	0.0135 (6)	0.0163 (6)	0.0031 (5)	−0.0021 (5)	−0.0008 (5)
C21'	0.0353 (9)	0.0205 (7)	0.0206 (7)	0.0008 (6)	−0.0035 (6)	−0.0049 (6)
C22	0.0160 (6)	0.0175 (6)	0.0151 (6)	0.0026 (5)	−0.0006 (5)	0.0028 (5)
C22'	0.0231 (7)	0.0207 (7)	0.0238 (7)	−0.0019 (6)	0.0068 (6)	0.0018 (6)
C23	0.0172 (7)	0.0152 (6)	0.0172 (6)	−0.0003 (5)	−0.0019 (5)	0.0020 (5)

### Geometric parameters (Å, º)

**Table:**

O1—C1	1.3647 (16)	C10—H10	0.9500
O1—C17	1.3936 (17)	C11—C12	1.409 (2)
O2—C5	1.2237 (17)	C11—H11	0.9500
O20—C20	1.3671 (16)	C12—C13	1.369 (2)
O20—C20'	1.4235 (17)	C12—H12	0.9500
O21—C21	1.3784 (15)	C13—C14	1.415 (2)
O21—C21'	1.4384 (17)	C13—H13	0.9500
O22—C22	1.3610 (16)	C14—C15	1.418 (2)
O22—C22'	1.4204 (16)	C15—C16	1.362 (2)
C1—C6	1.3431 (19)	C15—H15	0.9500
C1—C2	1.495 (2)	C16—C17	1.4105 (19)
C2—C3	1.523 (2)	C16—H16	0.9500
C2—H2A	0.9900	C18—C19	1.3884 (18)
C2—H2B	0.9900	C18—C23	1.3934 (19)
C3—C4	1.522 (2)	C19—C20	1.3972 (18)
C3—H3A	0.9900	C19—H19	0.9500
C3—H3B	0.9900	C20—C21	1.3926 (19)
C4—C5	1.511 (2)	C20'—H20A	0.9800
C4—H4A	0.9900	C20'—H20B	0.9800
C4—H4B	0.9900	C20'—H20C	0.9800
C5—C6	1.4698 (18)	C21—C22	1.3978 (19)
C6—C7	1.5136 (19)	C21'—H21A	0.9800
C7—C8	1.5174 (18)	C21'—H21B	0.9800
C7—C18	1.5342 (17)	C21'—H21C	0.9800
C7—H7	1.0000	C22—C23	1.3913 (18)
C8—C17	1.3681 (19)	C22'—H22A	0.9800
C8—C9	1.4361 (19)	C22'—H22B	0.9800
C9—C10	1.413 (2)	C22'—H22C	0.9800
C9—C14	1.4311 (19)	C23—H23	0.9500
C10—C11	1.376 (2)		
			
C1—O1—C17	117.86 (11)	C12—C13—C14	120.92 (14)
C20—O20—C20'	117.48 (11)	C12—C13—H13	119.5
C21—O21—C21'	112.96 (10)	C14—C13—H13	119.5
C22—O22—C22'	117.24 (11)	C13—C14—C15	121.18 (13)
C6—C1—O1	122.89 (13)	C13—C14—C9	119.47 (13)
C6—C1—C2	125.50 (13)	C15—C14—C9	119.34 (13)
O1—C1—C2	111.61 (12)	C16—C15—C14	120.89 (13)
C1—C2—C3	111.14 (12)	C16—C15—H15	119.6
C1—C2—H2A	109.4	C14—C15—H15	119.6
C3—C2—H2A	109.4	C15—C16—C17	118.90 (13)
C1—C2—H2B	109.4	C15—C16—H16	120.6
C3—C2—H2B	109.4	C17—C16—H16	120.6
H2A—C2—H2B	108.0	C8—C17—O1	122.82 (12)
C4—C3—C2	110.64 (13)	C8—C17—C16	123.67 (13)
C4—C3—H3A	109.5	O1—C17—C16	113.51 (12)
C2—C3—H3A	109.5	C19—C18—C23	120.43 (12)
C4—C3—H3B	109.5	C19—C18—C7	120.46 (12)
C2—C3—H3B	109.5	C23—C18—C7	119.03 (12)
H3A—C3—H3B	108.1	C18—C19—C20	119.66 (12)
C5—C4—C3	113.31 (12)	C18—C19—H19	120.2
C5—C4—H4A	108.9	C20—C19—H19	120.2
C3—C4—H4A	108.9	O20—C20—C21	115.12 (12)
C5—C4—H4B	108.9	O20—C20—C19	124.60 (12)
C3—C4—H4B	108.9	C21—C20—C19	120.28 (12)
H4A—C4—H4B	107.7	O20—C20'—H20A	109.5
O2—C5—C6	120.65 (13)	O20—C20'—H20B	109.5
O2—C5—C4	121.49 (12)	H20A—C20'—H20B	109.5
C6—C5—C4	117.81 (12)	O20—C20'—H20C	109.5
C1—C6—C5	119.46 (13)	H20A—C20'—H20C	109.5
C1—C6—C7	122.09 (12)	H20B—C20'—H20C	109.5
C5—C6—C7	118.45 (12)	O21—C21—C20	120.02 (12)
C6—C7—C8	109.41 (11)	O21—C21—C22	120.31 (12)
C6—C7—C18	112.10 (11)	C20—C21—C22	119.65 (12)
C8—C7—C18	109.24 (10)	O21—C21'—H21A	109.5
C6—C7—H7	108.7	O21—C21'—H21B	109.5
C8—C7—H7	108.7	H21A—C21'—H21B	109.5
C18—C7—H7	108.7	O21—C21'—H21C	109.5
C17—C8—C9	117.65 (12)	H21A—C21'—H21C	109.5
C17—C8—C7	119.91 (12)	H21B—C21'—H21C	109.5
C9—C8—C7	122.23 (12)	O22—C22—C23	125.04 (12)
C10—C9—C14	117.97 (13)	O22—C22—C21	114.79 (12)
C10—C9—C8	122.74 (12)	C23—C22—C21	120.15 (12)
C14—C9—C8	119.28 (13)	O22—C22'—H22A	109.5
C11—C10—C9	121.20 (13)	O22—C22'—H22B	109.5
C11—C10—H10	119.4	H22A—C22'—H22B	109.5
C9—C10—H10	119.4	O22—C22'—H22C	109.5
C10—C11—C12	120.53 (14)	H22A—C22'—H22C	109.5
C10—C11—H11	119.7	H22B—C22'—H22C	109.5
C12—C11—H11	119.7	C22—C23—C18	119.82 (12)
C13—C12—C11	119.90 (14)	C22—C23—H23	120.1
C13—C12—H12	120.1	C18—C23—H23	120.1
C11—C12—H12	120.1		
			
C17—O1—C1—C6	−13.87 (19)	C8—C9—C14—C15	−0.18 (19)
C17—O1—C1—C2	165.29 (11)	C13—C14—C15—C16	−177.73 (13)
C6—C1—C2—C3	−22.4 (2)	C9—C14—C15—C16	3.8 (2)
O1—C1—C2—C3	158.43 (12)	C14—C15—C16—C17	−2.9 (2)
C1—C2—C3—C4	48.01 (16)	C9—C8—C17—O1	−175.23 (11)
C2—C3—C4—C5	−52.63 (16)	C7—C8—C17—O1	10.04 (19)
C3—C4—C5—O2	−153.03 (13)	C9—C8—C17—C16	5.5 (2)
C3—C4—C5—C6	29.63 (18)	C7—C8—C17—C16	−169.20 (12)
O1—C1—C6—C5	177.52 (12)	C1—O1—C17—C8	9.55 (18)
C2—C1—C6—C5	−1.5 (2)	C1—O1—C17—C16	−171.14 (11)
O1—C1—C6—C7	−1.8 (2)	C15—C16—C17—C8	−2.0 (2)
C2—C1—C6—C7	179.13 (13)	C15—C16—C17—O1	178.70 (12)
O2—C5—C6—C1	−179.34 (13)	C6—C7—C18—C19	−120.36 (13)
C4—C5—C6—C1	−1.98 (19)	C8—C7—C18—C19	118.19 (13)
O2—C5—C6—C7	0.03 (19)	C6—C7—C18—C23	62.98 (15)
C4—C5—C6—C7	177.40 (12)	C8—C7—C18—C23	−58.47 (16)
C1—C6—C7—C8	19.27 (17)	C23—C18—C19—C20	0.7 (2)
C5—C6—C7—C8	−160.10 (11)	C7—C18—C19—C20	−175.94 (12)
C1—C6—C7—C18	−102.08 (15)	C20'—O20—C20—C21	−175.65 (12)
C5—C6—C7—C18	78.55 (14)	C20'—O20—C20—C19	4.98 (19)
C6—C7—C8—C17	−22.82 (16)	C18—C19—C20—O20	179.03 (12)
C18—C7—C8—C17	100.24 (14)	C18—C19—C20—C21	−0.3 (2)
C6—C7—C8—C9	162.70 (12)	C21'—O21—C21—C20	−105.78 (14)
C18—C7—C8—C9	−74.24 (15)	C21'—O21—C21—C22	76.08 (16)
C17—C8—C9—C10	176.82 (13)	O20—C20—C21—O21	2.79 (18)
C7—C8—C9—C10	−8.6 (2)	C19—C20—C21—O21	−177.81 (12)
C17—C8—C9—C14	−4.30 (18)	O20—C20—C21—C22	−179.06 (12)
C7—C8—C9—C14	170.30 (12)	C19—C20—C21—C22	0.3 (2)
C14—C9—C10—C11	0.4 (2)	C22'—O22—C22—C23	0.51 (19)
C8—C9—C10—C11	179.30 (13)	C22'—O22—C22—C21	−178.19 (12)
C9—C10—C11—C12	−0.4 (2)	O21—C21—C22—O22	−3.82 (18)
C10—C11—C12—C13	−0.4 (2)	C20—C21—C22—O22	178.04 (12)
C11—C12—C13—C14	1.2 (2)	O21—C21—C22—C23	177.42 (12)
C12—C13—C14—C15	−179.52 (14)	C20—C21—C22—C23	−0.7 (2)
C12—C13—C14—C9	−1.1 (2)	O22—C22—C23—C18	−177.55 (12)
C10—C9—C14—C13	0.3 (2)	C21—C22—C23—C18	1.1 (2)
C8—C9—C14—C13	−178.63 (13)	C19—C18—C23—C22	−1.1 (2)
C10—C9—C14—C15	178.75 (13)	C7—C18—C23—C22	175.60 (12)

### Hydrogen-bond geometry (Å, º)

*Cg*1, *Cg*2 and *Cg*3 are the centroids of the C8/C9/C14–C17, C18–C23 and C9–C14 rings, respectively.

**Table:**

*D*—H···*A*	*D*—H	H···*A*	*D*···*A*	*D*—H···*A*
C16—H16···O20^i^	0.95	2.36	3.2604 (18)	159
C2—H2*B*···*Cg*1^ii^	0.99	2.92	3.8088 (16)	150
C4—H4*B*···*Cg*2^iii^	0.99	2.75	3.5605 (16)	140
C22′—H22*B*···*Cg*2^iv^	0.98	2.56	3.3918 (16)	143
C22′—H22*C*···*Cg*3^iv^	0.98	2.78	3.4332 (16)	125

Symmetry codes: (i) −*x*+1/2, *y*−1/2, *z*; (ii) *x*−1/2, −*y*+1/2, −*z*; (iii) −*x*, −*y*+1, −*z*; (iv) *x*−3/2, *y*, −*z*−1/2.
